# Author Correction: The first demonstration of entirely roll-to-roll fabricated perovskite solar cell modules under ambient room conditions

**DOI:** 10.1038/s41467-024-47910-4

**Published:** 2024-04-26

**Authors:** Hasitha C. Weerasinghe, Nasiruddin Macadam, Jueng-Eun Kim, Luke J. Sutherland, Dechan Angmo, Leonard W. T. Ng, Andrew D. Scully, Fiona Glenn, Regine Chantler, Nathan L. Chang, Mohammad Dehghanimadvar, Lei Shi, Anita W. Y. Ho-Baillie, Renate Egan, Anthony S. R. Chesman, Mei Gao, Jacek J. Jasieniak, Tawfique Hasan, Doojin Vak

**Affiliations:** 1 Flexible Electronics Laboratory, CSIRO Manufacturing, Clayton, VIC 3168 Australia; 2 Cambridge Graphene Centre, University of Cambridge, Cambridge, CB3 0FA UK; 3 Department of Materials Science and Engineering, Monash University, Clayton, VIC 3800 Australia; 4 School of Materials Science and Engineering (MSE), Nanyang Technological University (NTU), 50 Nanyang Ave, Block N4.1, Singapore, 639798 Singapore; 5 School of Photovoltaic and Renewable Energy Engineering, University of New South Wales, Sydney, NSW 2052 Australia; 6 Foshan Xianhu Laboratory of the Advanced Energy Science and Technology Guangdong Laboratory, Foshan, China; 7 Sydney Nano and School of Physics, Faculty of Science, The University of Sydney, Sydney, NSW 2006 Australia

**Keywords:** Solar cells, Chemical engineering, Design, synthesis and processing

Correction to: *Nature Communications* 10.1038/s41467-024-46016-1, published online 12 March 2024

The original version of this article contained an error in Fig. 3c, in which the overlayed arrows and text moved behind the image.

The correct version of Fig. 3c is:
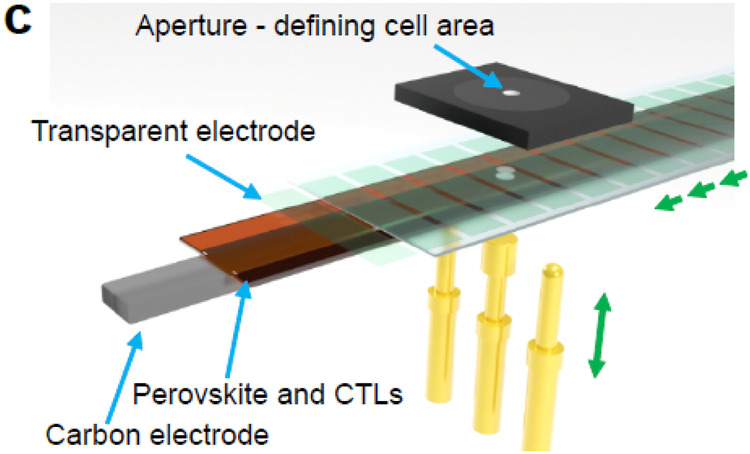


which replaces the previous incorrect version:
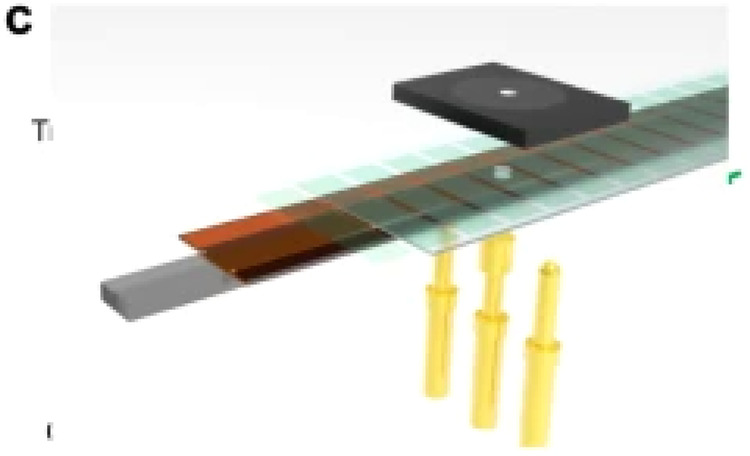


This has been corrected in both the PDF and HTML versions of the Article.

