# Supplementary material for: The first demonstration of entirely roll-to-roll fabricated perovskite solar cell modules under ambient room conditions
Source: Nat Commun. 2024 Mar 12;15:1656. doi: 10.1038/s41467-024-46016-1 (PMC10933357; doi:10.1038/s41467-024-46016-1)
Supplement: Supplementary file 1 — Supplementary Information [file 41467_2024_46016_MOESM1_ESM.pdf]

Supplementary Information for

# The first demonstration of entirely roll-to-roll fabricated perovskite solar cell modules under ambient room conditions

Hasitha C. Weerasinghe[1,8], Nasiruddin Macadam[2,8], Jueng-Eun Kim[1,3,8], Luke J. Sutherland[1,3], Dechan Angmo[1], Leonard WT Ng[1,2,4], Andrew D. Scully[1], Fiona Glenn[1], Regine Chantler[1], Nathan L. Chang[5], Mohammad Dehghanimadvar[5], Lei Shi[5,6], Anita WY Ho-Baillie[5,7], Renate Egan[5], Anthony S. R. Chesman[1], Mei Gao[1], Jacek J. Jasieniak[3]*, Tawfique Hasan[2]*, and Doojin Vak[1]*

Corresponding Authors: Doojin Vak (doojin.vak@csiro.au), Tawfique Hasan, (th270@cam.ac.uk), Jacek J. Jasieniak (jacek.jasieniak@monash.edu)

[1]Flexible Electronics Laboratory, CSIRO Manufacturing, Clayton, Victoria 3168, Australia

[2]Cambridge Graphene Centre, University of Cambridge, Cambridge CB3 0FA, UK

[3]Department of Materials Science and Engineering, Monash University, Clayton, Victoria 3800, Australia

[4]School of Materials Science and Engineering (MSE), Nanyang Technological University (NTU), 50 Nanyang Ave, Block N4.1, Singapore 639798, Singapore

[5]School of Photovoltaic and Renewable Energy Engineering, University of New South Wales, Sydney, NSW 2052, Australia

[6]Foshan Xianhu Laboratory of the Advanced Energy Science and Technology Guangdong Laboratory, Foshan, China

[7]Sydney Nano Institute and School of Physics, Faculty of Science, The University of Sydney, Sydney, NSW 2006, Australia

[8]These authors contributed equally: Hasitha C. Weerasinghe, Nasiruddin Macadam and Jueng-Eun Kim.

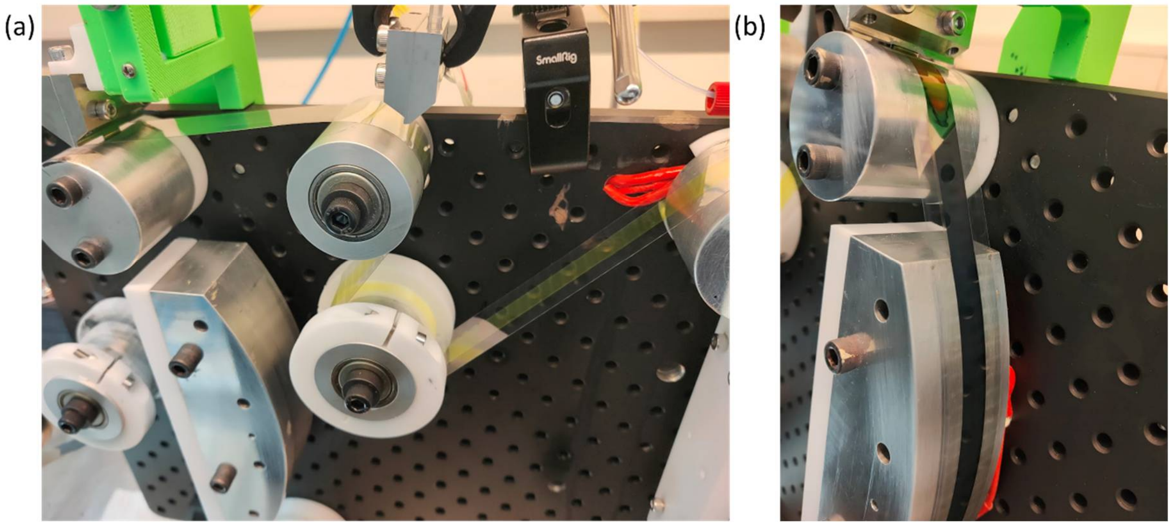


**Figure S1**. Roll-to-roll fabrication of perovskite films using the printing-friendly sequential deposition technique. (a) Formation of a glassy intermediate film with the edge-blowing technique and (b) instant conversion of the reactive intermediate to perovskite by MAI deposition. The perovskite film shows a reflected image of the breadboard demonstrating the smoothness of the film.

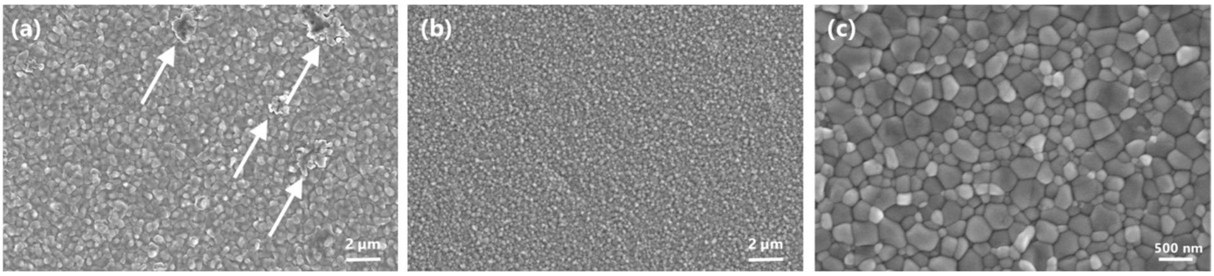


**Figure S2.** SEM images of perovskite films fabricated using the PFSD technique. (a) Surface of a sample with face blowing showing unreacted MAI (marked with arrows) on the surface. (b) and (c) Surface of the sample with edge blowing showing no sign of unreacted MAI.

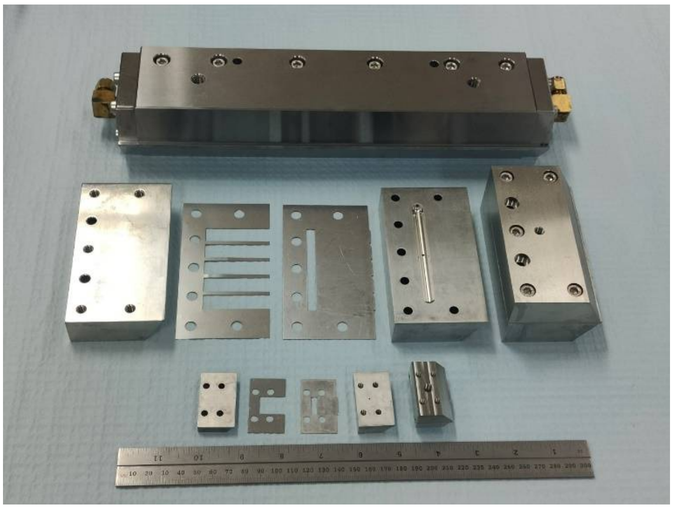

**Figure S3**. Slot die heads used for the fabrication of small cells (1st head block, shim, meniscus guide, 2nd head block with a solution inlet, and assembled head, from left to right) at bottom of the image and 10 cm-wide modules (1st head block, shim, meniscus guide, 2nd head block with a solution inlet, and assembled head, from left to right) in the centre of the image. An assembled 30 cm-wide head to be used for larger modules in the future is shown at the top of the image.

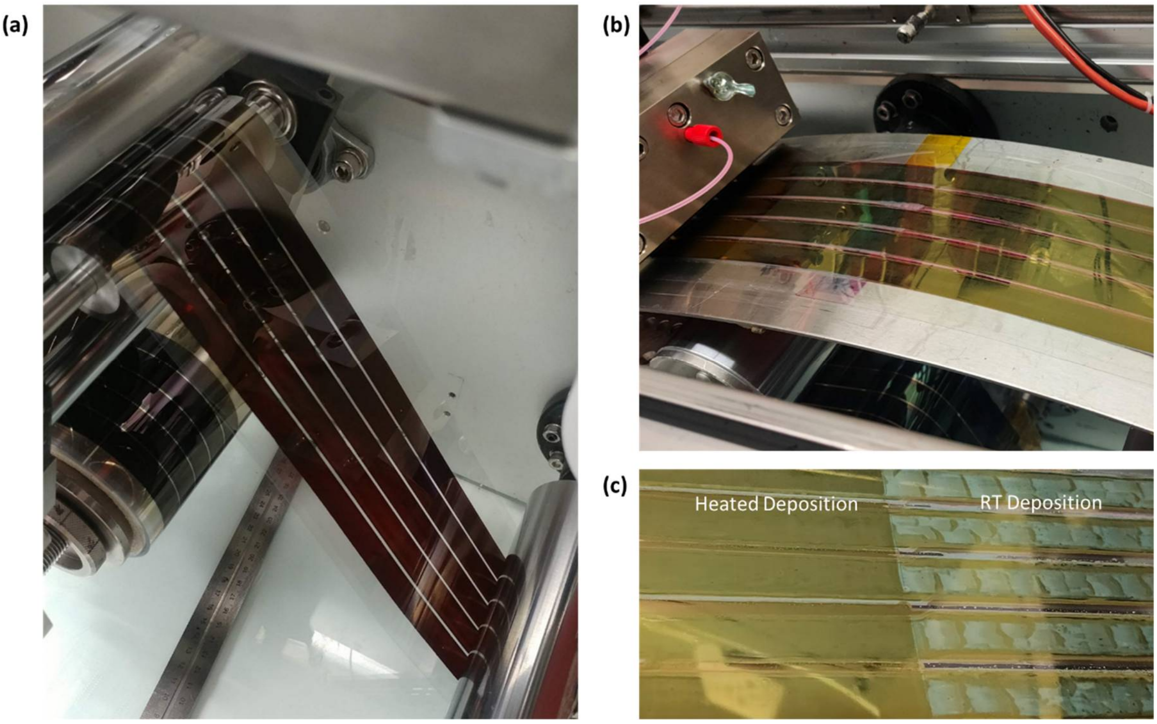


**Figure S4**. (a) A photograph of the high-quality large-area perovskite film fabricated by R2R SD coating, (b) SD coating of P3HT on a custom-built curved heating plate and (c) P3HT stripe fabricated at RT and 45°C.

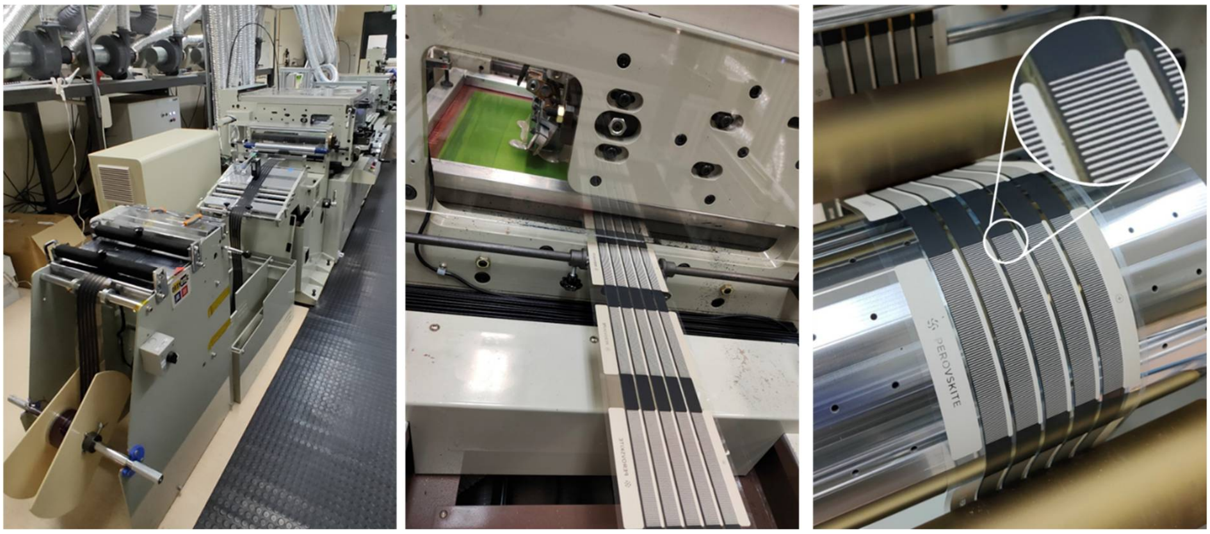


**Figure S5**. Additional photos of the roll-to-roll screen printing of metal grids and contacts of perovskite PV modules.

**Table S1**. Record efficiencies of flexible PeSCs fabricated by various processes.

| Category | Publicaion Year | Back Electrode | *PCE* (%) @ area (cm$^2$) | Reporting organisations | Ref. |
|---|---|---|---|---|---|
| Batch process[a] | 2017 | Vacuum-aluminium | 17.3 @ 0.018 | SNU, SKKU | 1 |
| | 2018 | Vacuum – gold | 18.4 @ 0.052 | SNNU, iChEM CAS, Virginia Tech | 2 |
| | 2019 | Vacuum - silver | 19.5 @ 0.09 | CSU, U. Rochester. NCNST CAS | 3 |
| | 2020 | Vacuum - silver | 19.9 @ 1.01 | Nanchang U., ICCAS, DHU, JXNU, CSU | 4 |
| | 2021 | Vacuum - silver | 21.7 @ 0.10 | CityU, SIAT CAS, HKU, U. Washington | 5 |
| | 2022 | Vacuum - gold | 23.6 @ 0.10 | Tsinghua U., NCNT CAS, ZHAW | 6 |
| | | | | | |
| Hybrid (R2R + batch) | 2018 | Vacuum - gold | 14.1 @ 0.15 | NREL, SLAC NAL, Corning, CU Boulder, | 7 |
| | 2020 | Vacuum - gold | 16.7 @ 0.096 | KRICT, CNU, VTT | 8 |

| | | | | | |
|---|---|---|---|---|---|
| R2R excluding electrodes | 2017 | Vacuum - Ag | 11.0 @ 0.10 | CSIRO, GIST, U. Melbourne | [9] |
| | 2018 | Vacuum - calcium/aluminium | 11.2 @ 0.10 | CSIRO, NCNT CAS, U. CAS | [10] |
| | 2019 | Vacuum - calcium/aluminium | 11.7 @ 0.10 | GIST, CSIRO, Kunsan NU, NCNT CAS | [11] |
| | 2020 Apr | Vacuum - silver | 12.2 @ 0.09 | Swansea U. | [12] |
| | 2020 Oct | Vacuum - gold | 13.8 @ 0.096 | KRICT, CNU, VTT | [8] |
| | 2022 | Vacuum - gold | 17.4 @ 0.08 | CSIRO, Monash U., | [13] |
| | 2023 | Vacuum - gold | 17.9 @ 0.08 | CSIRO, U. Cambridge, Monash U., NTU, UNSW, USYD | This work |
| Fully R2R | 2023 | R2R coating - Carbon | 10.8 @ 0.09 | Swansea U., | |
| | 2023 | R2R coating - Carbon | 15.4 @ 0.025 | CSIRO, U. Cambridge, Monash U., NTU, UNSW, USYD | [14] |
| Fully R2R - Module | 2023 | R2R coating - Carbon | 11.0 @ 49.5 | CSIRO, U. Cambridge, Monash U., NTU, UNSW, USYD | This work |

[a]There are a large number of publications on batch-processed flexible PeSCs. Therefore, only one record is listed for each year.

**Supplementary Note 1 – Fabrication of $SnO_2$ nanoparticle film on commercial TCEs.**

The slot-die (SD) coating of water-based $SnO_2$ nanoparticle inks did not reliably wet untreated flexible substrates well. We found the wetting to be extremely sensitive to the surface condition of the commercially produced TCEs, with dewetting occurring intermittently depending on the substrate batch and even the position on the rolls. It was impractical to eliminate this issue by cleaning the rolls of flexible films using methods typically employed in laboratories at a small scale, such as UV-ozone or vacuum-based $O_2$ plasma treatments. (An AR/$N_2$ plasma treatment has been successfully used with R2R gravure printing [15], but this treatment method is not available in our printing system.) A corona treatment was also attempted but some edges of the TCE patterns were damaged, resulting in a large deviation in device performance.

Given these problems, a precursor-based route[16] became the preferred approach to deposit $SnO_2$ thin films via R2R coating. A reverse gravure[17] (RG) method also improved wetting since the coating roll makes physical contact with the substrate and rubs the surface, which may create anchoring points for the inks. Therefore, RG coating was used for $SnO_2$ deposition.

While the RG method was useful in preparing the $SnO_2$ layer reliably, this coating system has an intrinsic issue with prolonged operation for some inks due to the open solvent reservoir system. Consequently, we found that this system is not suitable for the production of long films (50 – 100 m). Therefore, an additive-based approach was also explored, with the addition of a small amount of acetic acid (as described in the experimental section) found to be helpful in improving wetting without changing the properties of the produced films as the volatile chemical was removed during the annealing process. Therefore, both approaches were used depending on the deposition requirements of the experiments.

**Supplementary Note 2- Infrared boosts efficiencies within the time scale for R2R**.

Thermal annealing is necessary during the fabrication of PeSCs, but may also have a negative effect on device performance, especially when the process is performed in air. Infrared (IR) heaters are a commonly used alternative to conventional hot plates/air-blowers in high-throughput industrial printers for the drying or curing of inks for multiple reasons. IR heaters can reduce the annealing time for perovskite films from 45 min to 2.5 s[18] and can also selectively heat the perovskite layer without damaging the underlying plastic substrate.

Therefore, we investigated the use of IR heating to reduce the long thermal annealing time typically required for formamidinium (FA)-based formulations. It was found that IR annealing was not only fast but also effective for the complete conversion of the precursor used in the printing-friendly sequential deposition (PFSD) technique process. Hence, we tested IR-assisted perovskite conversion under various conditions using built-in IR heating units in an industrial screen printer used for module production. Four intensities were chosen and labelled as IR25, IR30, IR35, and IR40. The labels were made following the settings on the IR controller; IR25 was the minimum power required to light up the IR lamps and IR40 was the highest intensity for the IR treatment that could be used without damaging the plastic substrate, with IR30 and IR35 falling in between. The corresponding measured intensities for IR25, IR30, IR35, and IR40 were about 0.8, 1.4, 2.2, and 3.0 W/cm², respectively. All samples prepared in the same R2R batch were passed through the oven under IR lamps at 0.5 m/min speed (about 3 min exposure) under these conditions.

Figure S6 shows the results of the IR-treated samples. The yellow intermediate film prepared from the non-stoichiometric formulation quickly turns into a dark brown film even without any annealing, with the subsequent thermal annealing inducing further conversion, as evidenced by increased absorbance in the 600-800 nm range (shown in Figure S6a). The IR25 sample shows only a minor change, but IR30 or higher intensities show significant changes with improved conversion, which is beneficial to device performance. This result shows that IR is an alternative to thermal annealing, and may in fact deliver superior performance.

Measurements of the photoluminescence (PL) decay of the perovskite layer of PET/ITO/$SnO_2$/perovskite samples (Figure S6b) show that the thermally-annealed sample displays a faster PL decay than the unannealed sample, implying that thermal annealing induces a greater defect density in the perovskite layer, resulting in an increased non-radiative decay rate. In contrast, the IR-treated samples all display slower PL decay than the unannealed sample, suggesting a reduced defect density, with the longest-lived PL measured for the sample exposed to the IR35 condition. Although the perovskite absorbance upon IR40 treatment results in a stronger UV-visible absorption than the IR35, implying a more complete conversion to the perovskite in the bulk film, the higher intensity IR treatment also appears to create more defects in the perovskite layer.

As expected, the IR35 sample showed improved performance compared to the control device fabricated with thermal annealing, as shown in Figure S6c. IR treatment improved current density ($J_{sc}$) with minor improvement in open circuit voltage ($V_{oc}$) and fill factor (FF). The thermally annealed device showed 15.9% *PCE* with 20.0 mA/cm² $J_{sc}$, 1.02 V $V_{oc}$, and

77.4% *FF*. The IR35 device showed 17.9 % *PCE* with 21.9 mA/cm² $J_{sc}$, 1.04 V $V_{oc}$, and 78.4% *FF*. This improvement is attributed to the synergistic effect of reduced defect density and improved conversion. In contrast, the IR40 device shows only 52.7% *FF* caused by significantly increased series resistance (Rs) which would be caused by decomposition of the perovskite on the surface of the film. The device parameters were obtained from reverse scan (from open circuit to short circuit). The best device was tested further with forward scan and maximum power point (MPP) tracking methods, as shown in Figure S6d. The device showed 16.4% *PCE* with 21.7 mA/cm² $J_{sc}$, 1.04 V $V_{oc}$ and 72.6 % *FF*. The efficiency from the forward scan was consistent with the *PCE* obtained from MPP tracking after 100 sec.

Although this approach was found to be a promising means to achieve high-performance vacuum-based PV, we found the approach was not applicable to carbon-based devices, as thermal annealing is an essential part of the printing process. Therefore, the approach was not further investigated, however, the efficiency was used to calculate the energy cost of vacuum-based PV modules.

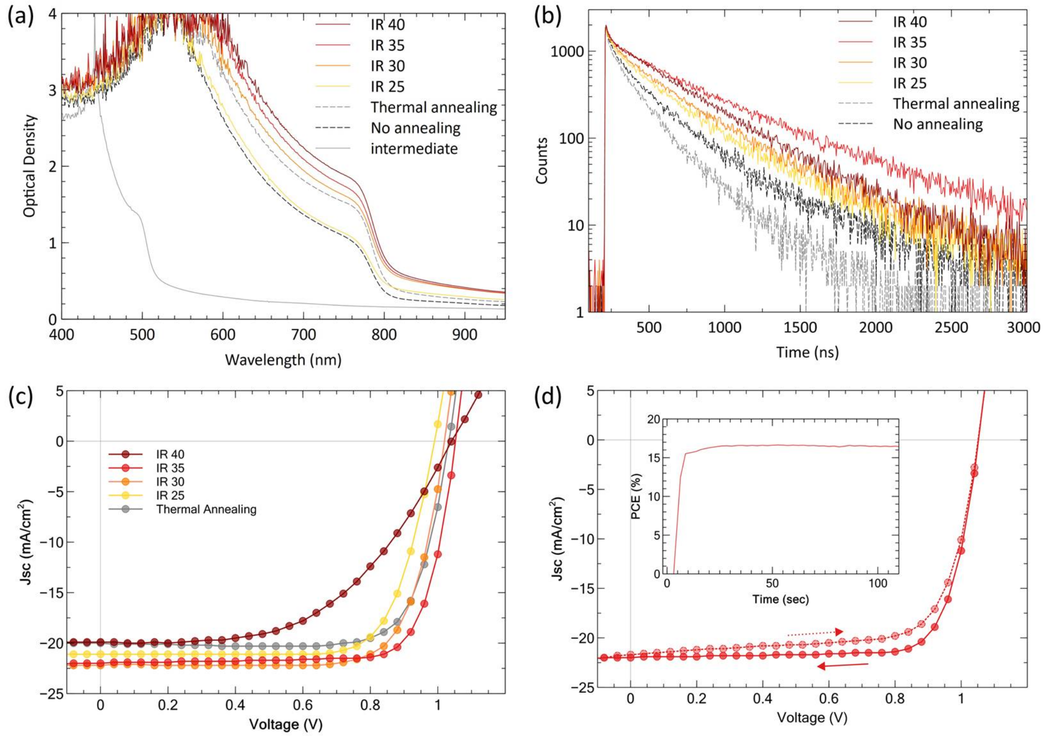


**Figure S6**. (a) UV-visible optical density spectra and (b) PL decay curves of $FA_{0.45}MA_{0.55}PbI_3$ perovskite films under various annealing conditions. (c) J-V curves of R2R-fabricated perovskite cells with various annealing conditions. Device configuration: flexible TCE / $SnO_2$ / $FA_{0.45}MA_{0.55}PbI_3$ / Spiro-OMeTAD / Au. (d) Forward and reverse J-V scans of the best device. Inset shows 16.4% *PCE* from the MPP tracking curve for 100 sec.

**Supplementary Note 3- Transition from vacuum deposition to printing**

PeSCs with printed back electrodes have been reported previously, but most of them are not suitable for R2R fabrication due to their high processing temperatures (> 140 °C) [19–22]. Most of them have a mesoporous $TiO_2$- or dense sintered $TiO_2$ layer, which can effectively prevent shunt path formation by solution-processed conductors. There are some examples of flexible devices with carbon electrodes, but their efficiency was very low (4% *PCE*)[23] or a vacuum process was used as well.[24] This could be due to the poorer charge-blocking performance of low-temperature-processed hole blocking and electron transport layers, which also prevent short circuits.

Therefore, we developed bespoke formulations of low-temperature-processable carbon pastes/inks. A key requirement was chemical compatibility with underlying layers. A HTL may or may not be required when carbon is used as an electrode, however, preliminary results showed poorer performance and reproducibility in HTL-free devices. Therefore, we focused on device configurations that included a HTL and investigated solvents that are compatible with both conjugated polymer and perovskite layers. Spiro-OMeTAD was found to be not suitable for printed electrodes due to its low thermal durability as thermal annealing over at least 120°C is essential to dry carbon and silver inks. At that stage, poly(3-hexylthiophene) (P3HT) was not an option as we previously found that P3HT alone does not perform as well as some other available HTLs[9]. We also tried commercially available HTL solutions developed for PeSCs such as Clevios HTL Solar 3 and Solar 4. These HTL solutions are commonly used poly(3,4-ethylenedioxythiophene) (PEDOT) dissolved in a perovskite compatible organic solvent (toluene for Solar 3 and anisole for Solar 4). Solar 3 was first reported by Hou et al.[25] and used in a recent report on the fully R2R fabricated PeSCs[14]. We also tested both materials and found they work better than P3HT, however, we also found poly[(2,5-bis(2-hexyldecyloxy)phenylene)-alt-(5,6-difluoro-4,7-di(thiophen-2-yl)benzo[c][1,2,5]-thiadiazole)] (PPDT2FBT) slightly better than the commercial HTL solutions.

PPDT2FBT was first developed for organic solar cells[26] and was found to be extremely thickness tolerent[27]. Despite its complex molecular structure, intramolecular hydrogen bonds lock the planarity of aromatic units and enhance molecular packing, which ensures good charge transportation performance. Therefore, the material has been regarded a printable material (sold as "Solar Polymer for R2R Printing") and we confirmed suitability for R2R printing by demonstrating record-breaking efficiency[28] (at the time of the publication) for R2R fabricated organic solar cells. The polymer was also used as an HTL and has shown high performance and

durability in PeSCs[29]. Therefore, we chose the polymer as a standard material while developing other parts of PeSCs.

A number of aliphatic solvents with medium polarity were chosen for the formulation of conductive pastes/inks. We screened the performance of three solvents; diethylene glycol butyl ether (DGBE), propylene glycol methyl ether acetate (PGMEA) and α-terpineol. Ethyl cellulose is a non-toxic, water-insoluble polymeric binder widely used in the coating and printing industries.[30,31] Preliminary tests showed good compatibility of EC with perovskites and so it was selected as the binder for the ink. A 1:1 mixture of carbon powders (carbon black (Vulcan XC72, Cabot) and graphene nanoplatelet powder (G3 graphene, Levidian Nanosystems)) was used as the conductive pigment in the formulation predominantly to establish optimal conductivity with the lowest effect on viscosity. Using the formulation of a previous ink as a base,[30] we began formulating inks with a carbon pigment content of up to 30 wt.%. Three-roll milling, an established dispersion technique for high-viscosity inks, was used to break down the large agglomerates of the carbon powders and disperse them within the solvent/binder matrix. The sheet resistances ($R_s$) of 25 μm (wet) film of the screen-printing inks are 80 Ω/□, 140 Ω/□ and 50 Ω/□ for DGBE, terpineol and PGMEA-based inks respectively.

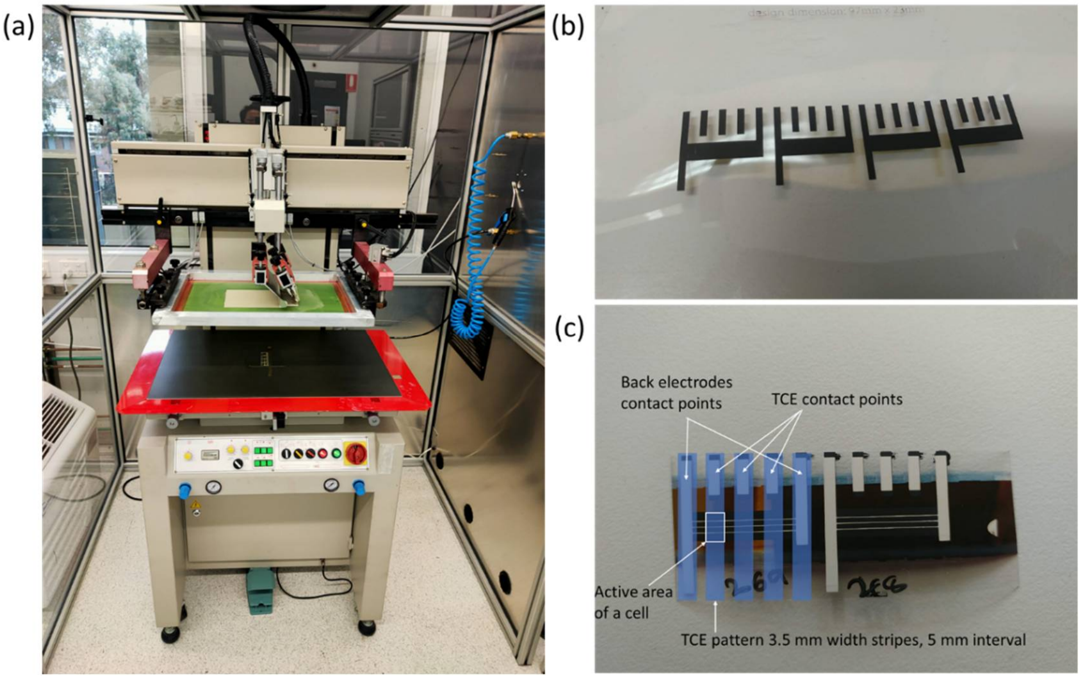


**Figure S7**. (a) The screen printer used to producethe carbon electrode and silver grids of vacuum-free PeSCs. (b) The screen  printed carbon layer used in the vacuum-free devices. (c) The device layout of flexible solar cells used to screen carbon pastes. ETL, perovskite layer, and HTL were roll-to-roll fabricated. Carbon and silver pastes were sequentially screen printed after cutting into 10 cm length.

Three pastes were formulated, using DGBE, α-terpineol and PGMEA, the latter of which has been used successfully in PeSCs with a high-temperature ETL.[20] We also explored several commercial carbon pastes that have been used successfully in glass-based PeSCs. [22,32] The carbon pastes were tested by printing carbon electrodes on top of R2R-fabricated PeSC films. [2928]The pastes were previously tested while optimizing the deposition conditions of the active layer, but all devices were fabricated again, allowing for a fair comparison using the same batch of perovskite films. A 10+ m length of the film (flexible TCE/ETL/perovskite/HTL) was R2R fabricated, and then cut into 10 cm strips onto which carbon and silver electrodes were screen printed using a semi-automatic flatbed screen printer, as shown in Figure 7a. The layout of the carbon pattern (Fig. S7b) and the overall device layout (Fig. S7c) are also shown.

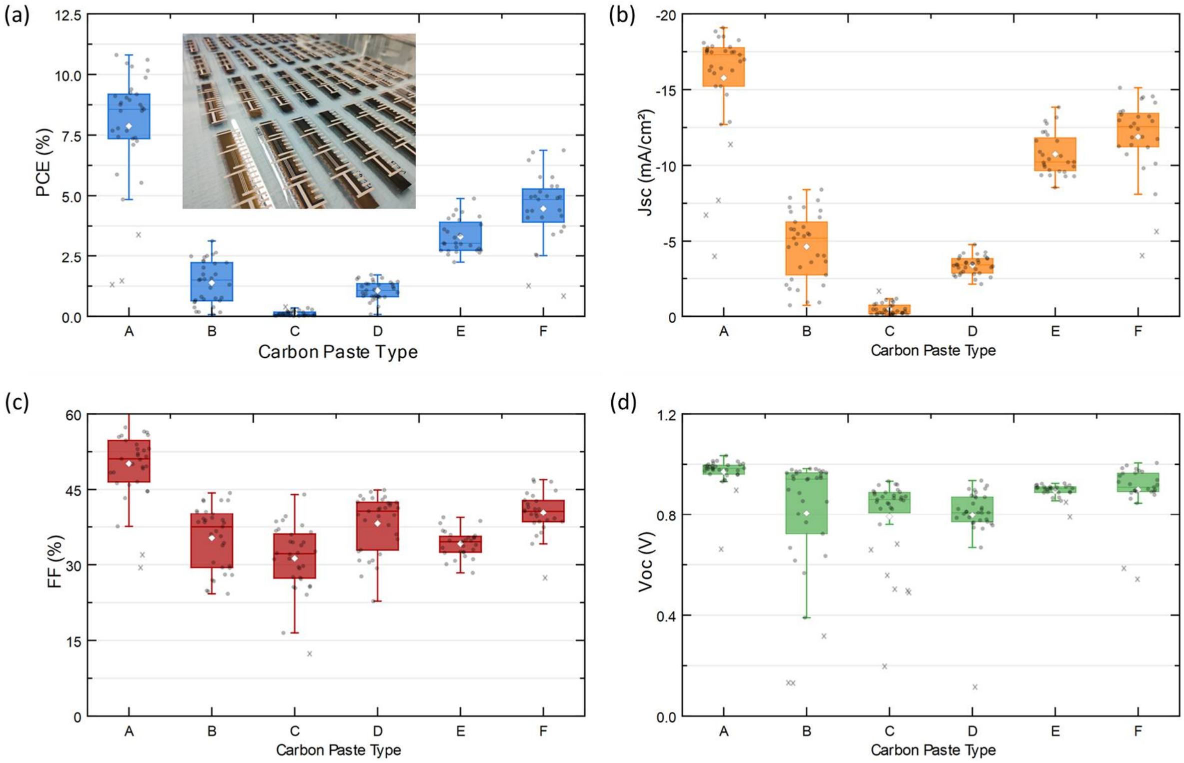


**Figure S8**. (a) *PCE* (inset shows a photograph of printed PeSCs used for carbon-paste screening), (b) $J_{sc}$, (c) $V_{oc}$, and (d) *FF* of printed PeSCs with a device configuration of flexible TCE / $SnO_2$ / $FA_{0.45}MA_{0.55}PbI_3$ / PPDT2FBT / screen printed carbon /screen printed silver (Dupont PV 416). A: PGMEA-based, B: DGBE-based, C: Terpineol-based, D: commercial (DongDaLai), E: commercial (Dycotec), F: commercial (Jelcon). The error bars represent 1.5 times the interquartile range.

Figure 8 shows the device parameters of the printed PeSCs with various carbon pastes. The PGMEA-based paste (label A) clearly outperformed while the terpineol-based paste

showed the worst device performance. The biggest performance loss was from $J_{sc}$ while $V_{oc}$ was largely maintained. When conductive defects are formed through the active layer, devices show a voltage loss. Therefore, the major loss is attributed to solvent damage to the HTL and/or top surface of the perovskite layer.

For further insight, work function levels of the pastes were investigated and the results are shown in Figure 9. Interestingly, the best-performing paste showed the least ideal work function with an order of magnitude higher resistivity than commercial ones. The result shows the importance of the chemical compatibility of the solvent with underlying layers. Furthermore, the different solvents led to a different work function of the carbon layer even though they were made with carbon formulations with the same solid components. These results shed light on future research directions for the printable electrodes for PeSCs. The PGMEA-based formulation has room for further improvement in terms of work function and conductivity. Further optimization of the formulation would lead to higher-performance vacuum-free PeSCs, more comparable to vacuum-based ones.

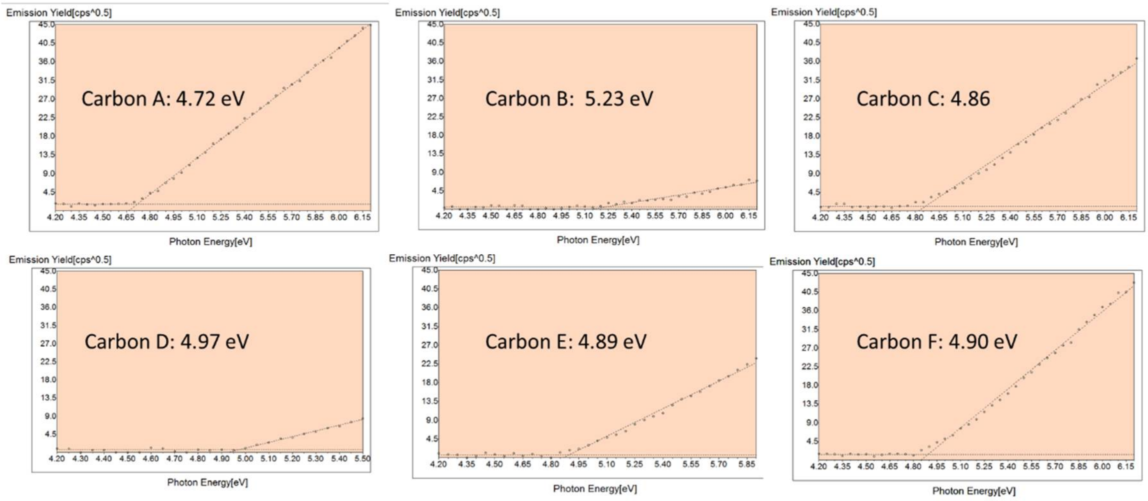


**Figure S9**. Photoelectron spectroscopy in air (PESA) results for carbon pastes used in this work. A: Bespoke carbon with PGMEA, B: bespoke carbon with DGBE, C: bespoke carbon with Terpineol, D: commercial paste (DongDaLai), E: commercial paste (Dycotec, perovskite grade), and F: commercial paste (Jelcon).

The PGMEA-based formulation was found to deliver the highest performance, but it was found to be unsuitable for prolonged operation of screen printing due to the volatility of the solvent (bp: 146 °C). Therefore, we targeted an ink with lower viscosity (<1 Pa.s) that would be suitable for SD coating or RG coating. These methods supply fresh ink during roll-to-roll coating and are therefore more suitable for use with volatile solvent-based inks. We achieved

this by diluting the high-viscosity ink with a binder solution in the same solvent. For a 500 g batch of ink, we mixed 40 g of EC in 330 g of PGMEA and stirred until the binder was completely dissolved (Fig. S10a). 100 g of the solution was kept aside for a subsequent step in the formulation process. 130 g of the conductive carbon pigment was added to the remaining 270 g of solution to form a slurry (Fig. S10b). This mixture was processed through a three-roll mill yielding a uniform high viscosity ink (Fig. S10c). The remaining 100 g of EC/PGMEA was mixed into to the high viscosity ink by hand and then stirred using a magnetic stir bar until a uniform ink was formed (Fig. S9d). The starting viscosity of this ink under zero shear is ~0.15 Pa.s. We found that the optimal carbon content in our final ink was 26 wt.% with a binder content of 8 wt.%. The PGMEA-based carbon ink was then used with SD coating to fabricate PeSCs and the device was further optimized.

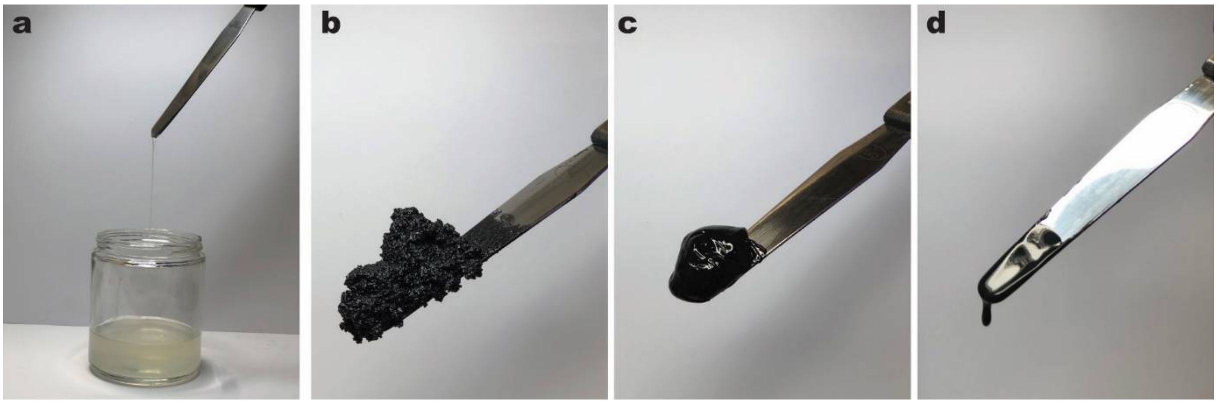


**Figure S10**. Different stages of ink formulation: a) EC/PGMEA solution, b) slurry after carbon pigment is added, c) after processing in the three-roll mill, d) the final ink.

Since the carbon paste alone was not conductive enough to be used as an electrode, a silver paste was screen printed to form charge collection grids and create contact points. Figure 1 shows the device performance of carbon-based PeSCs with different silver electrode patterns. Obviously, no-grid cells showed poor performance due to the low conductivity of the carbon-only cells. While the grids are clearly beneficial, a higher coverage of the silver pattern also induced degradation of the device performance regardless of the higher conductivity through the grids. The degradation is attributed to solvent damage to the underlying layers. This problem may be solved by replacing the solvent-based paste with a solventless curable silver paste. However, this approach was not tried due to the absence of a UV curing station in the R2R screen printer used for the production of the PeSC modules. Instead, an automatic testing setup was made to test carbon-based small cells without requiring the silver grids, as discussed in the

main text. For large-area modules, grids were designed to be the same as the 0.2 mm grid to achieve the required conductivity with minimum solvent damage.

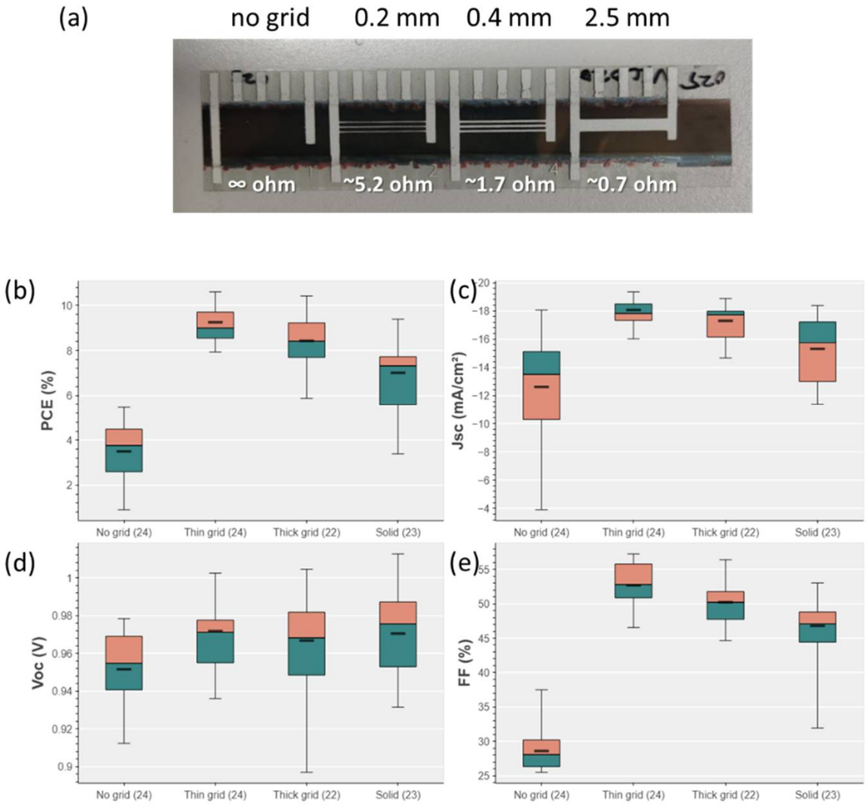


**Figure S11**. (a) Screen printed silver electrode designs. The no-grid design was used to test the carbon only electrode (~ 800 Ω sq$^{-1}$). The resistances in the figure shows resistances measured between side bus bars through the grids. (b) *PCE*, (c) $J_{sc}$, (d) $V_{oc}$, and (e) *FF* of printed PeSCs with the various printed silver electrode designs.

### ***Supplementary Note 4 – Cost Model Details***

As discussed, the cost model used in this study is based on our previous work.[33] The key change is the production speed used in the model. Previous work used only a demonstrated coating speed of 0.2-0.3 m/min and such low speeds are unlikely to be used in commercial factories. Therefore, we changed the production speed to be more realistic, 5 m/min, regardless of the coating speeds used in this research work. The speed should be achievable for all processes, however, the encapsulation (lamination) process may need a time-consuming preconditioning process which is required to prevent moisture/oxygen outgassing from barrier

materials.[34] The preconditioning may or may not be required depending on the moisture/oxygen tolerance of the modules and this process was not considered in the model. We also used an 85% geometric fill factor (GFF), expected upper limit[35] and a realistic number for commercial products fabricated by industrial printers, rather than the 75% GFF demonstrated in this work with the lab machine with a basic web guide system.

In brief, bottom-up assumptions for equipment purchase and running costs, labour, materials cost and usage were used to calculate the cost using a cost-of-ownership approach. Due to uncertainties in the exact values for each of these inputs, each input was given a range of values based on the information available to the authors. These are parameterized as being Low, Med or High in value. To account for these uncertainties, a Monte Carlo approach was taken, where the cost calculations were carried out many (in this case 50,000) times. For each calculation (or iteration), every input variable was given a value by independently sampling a random distribution that is defined by the Low, Med and High values. In this case, a two-half log-normal distribution was used, where Low = 10$^{th}$ percentile, Med = 50$^{th}$ percentile and High = 90$^{th}$ percentile. This allows for values both lower than the low and higher than the high estimate. The log-normal distribution avoids negative values at the lower end of the distribution. For each iteration, the manufacturing cost was calculated, thus providing 50,000 different cost calculations. The distribution of the cost results indicated the most likely range of costs for manufacturing each sequence, based on the estimates and uncertainties of the underlying input assumptions.

**Table S2**. Overall assumptions for the factory based in Australia.

| **Parameter** | **Range of values** |
|---|---|
| Annual factory production (m2/year) | 1 million $m^2$/year |
| Building construction rate (USD/m2) | 956 (849 – 1034) |
| Depreciation time (equipment and facilities) | 7 years |
| Depreciation time (building shell) | 15 years |
| Operator hourly rate USD/h | 25 |
| Technician hourly rate USD/h | 36 |
| Electricity price USD/kWh | 0.16 (0.17 – 0.22) |

*Note: assume facility requires 1kWh of electricity for every 1kWh of electricity consumed by production equipment.*

*Assume that there is an additional cost of 10% of direct labour for indirect labour.*

*Factory operates 24h/day, 365 days/year.*

Table S3 shows the processes assigned to each sequence, together with the associated equipment and materials.

**Table S3**. Definition of modelled process steps for sequences “A-Vacuum electrode”, “B- Printed electrode” and “C- commercial TCE-free and silver grid-free”.

| A | B | C | Process Description | Equipment | Major Materials |
|---|---|---|---|---|---|
| X | X | | Patterned OPV8 based substrate | | TCE |
| | | X | PET substrate | | PET substrate |
| X | X | X | R2R Frame Equipment | R2R Coating Frame | |
| | | X | ITO free – in-house | R2R coater – single coating add-on | PEDOT:PSS |
| X | X | X | ETL | R2R coater – single coating add-on | $SnO_2$ NPs |
| X | X | X | two-step perovskite layer | R2R coater – 2 single coating add-on | DMF, IPA, FAI, MAI, $PbI_2$ |
| X | | | HTL for vacuum silver | R2R coater – single coating add-on | FK 209 Co(III) $PF_6$ salt, LiTFSi, Spiro, TBP |
| | X | | HTL for Carbon | R2R coater – single coating add-on | HTAB, P3HT, chlorobenzene |
| | X | X | Carbon Electrode | R2R coater – single coating add-on | Ethyl cellulose, Graphite, PGMEA |
| | | X | Carbon Contact | R2R Screen printer | Commercial carbon paste |
| X | | | Gold evaporation | R2R evaporator for gold | Au pellets |
| | X | | Silver grid Contact | R2R screen printer | Ag paste |
| X | X | X | Module encapsulation (with 2 barrier films) | Laminator | Adhesive |

The cost data used to calculate equipment depreciation, running costs and labour are shown in Tables S4 and S5.

**Table S4**. Equipment cost, floor space and electricity assumptions

| Equipment | Cost US$k | Floor Space ($m^2$) | Electricity (kW) |
|---|---|---|---|
| R2R Coating Frame | 59.0 (36.0 – 198.0) | 3.0 (2.0 – 4.0) | 6.0 (4.0 – 9.0) |
| Roll to roll coater – single coating add-on | 28.0 (17.0 – 92.0) | 1.5 (1.0 – 2.0) | 6.0 (4.0 – 9.0) |
| R2R Screen printer | 67.0 (51.0 – 86.0) | 10.0 (8.0 – 15.0) | 15.0 (10.0 – 20.0) |
| Roll to roll evaporator for gold | 1500.0 (1500.0 – 2000.0) | 60.0 (50.0 – 90.0) | 840.0 (400.0 – 1000.0) |
| Laminator | 28.0 (22.0 – 36.0) | 10.0 (8.0 – 15.0) | 25.0 (20.0 – 30.0) |

**Table S5**. Equipment staff and throughput data

| Equipment | #Staff (Operation) | #Staff (Technician) | Throughput | Down Time (%) |
|---|---|---|---|---|
| R2R Coating Frame | 0.5 (0.25 – 1.0) | 2.0 (1.0 – 3.0) | 5.0 (m/min) | 10.0 (5.0 – 20.0) |
| Roll to roll coater – single coating add-on | 0.5 (0.25 – 1.0) | 2.0 (1.0 – 3.0) | 5.0 (m/min) | 10.0 (5.0 – 20.0) |
| R2R Screen printer | 0.05 (0.02 – 0.1) | 2.0 (1.0 – 3.0) | 5.0 (m/min) | 10.0 (5.0 – 20.0) |
| Roll to roll evaporator for gold | 0.25 (0.2 – 0.5) | 2.0 (1.0 – 3.0) | 1.0 (m/min) | 3.0 (2.0 – 5.0) |

| | | | | |
|---|---|---|---|---|
| Laminator | 0.02 (0.01 – 0.04) | 2.0 (1.0 – 3.0) | 90.0 ($m^2$/h) | 2.0 (1.0 – 5.0) |

Material usage was estimated based on lab experience of the material dosing rate in slot die coating combined with the web speed. Since spacing is required between strips to allow for interconnection, a ratio between material coverage and active area of 0.9 was assumed. In addition, to account for border areas, a ratio between active area and module area of 0.85 was assumed.

Materials costs at high manufacturing volume are particularly difficult to estimate, since the amount purchased is so much larger than that typically purchased for lab development purposes. Materials suppliers are unwilling to provide pricing at volume to university and institute researchers, and in some cases materials are not yet manufactured in large volumes, so pricing data is not known.

To account for these uncertainties, we use a volume pricing model which is based on a logarithmic relationship between purchase volume and price. We start with the pricing and purchase amounts that are available to our groups. We then estimate (with uncertainty ranges) a doubling factor D, which is the multiplier for the cost every time the purchase volume doubles. Mathematically, this is calculated with the following equation 1 and equation 2:

$$N_i = log_2\left(\frac{V_i}{V_k}\right) \qquad (1)$$

$N_i$ = the calculated number of doublings of purchase volume between the known volume and and factory annual volume for iteration i.

$V_i$ = the annual purchase volume for iteration i, based on process usage and factory annual throughput for iteration i.

$V_k$ = the purchase volume for the known purchase price

$$P_i = P_k * D_i^{N_i} \qquad (2)$$

Where

$P_i$ = the calculated purchase price for iteration i

$P_k$ = the known purchase price per unit

$D_i$ = the doubling factor for iteration i

In the previous work, the uncertainty range of the doubling factor was Low = 0.85, Med = 0.90, High = 0.95 for every material item. For this work, a different range was used for different materials based on research into volume pricing. The values for doubling factor and known pricing are shown in Table S6.

**Table S6**. Material cost assumptions. Pk = known price, Vk = purchase volume for known price. The doubling factor range was based on observations from data collected by the authors, but in each case the high value of the range was 0.95 to account for the possibility that observed cost reductions do not extrapolate to manufacturing volumes.

| Material | Material Unit | Pk | Vk | Doubling factor range |
|---|---|---|---|---|
| TCE | m2 | 103 | 1500 | 0.9 (0.85 - 0.95) |
| Methanol | ml | 0.047 | 4000 | 0.8 (0.65 - 0.95) |
| PEDOT | ml | 0.51 | 1000 | 0.85 (0.8 - 0.9) |
| PET substrate | m2 | 1.4 | 1000 | 0.85 (0.65 - 0.95) |
| Acetic acid | µl | 0.000645 | 100000 | 0.82 (0.65 - 0.95) |
| DI water | ul | 2.63E-09 | 1E+09 | 0.82 (0.65 - 0.95) |
| SnO (commercial) | mg | 8.83E-05 | 2000000 | 0.85 (0.65 - 0.95) |
| Ethanol | ul | 4.01E-05 | 2E+08 | 0.87 (0.65 - 0.95) |
| Ethylenediamine | ul | 2.79E-05 | 2500000 | 0.65 (0.65 - 0.95) |
| KOH | mg | 6.07E-05 | 2500000 | 0.78 (0.65 - 0.95) |
| SnCl4 | mg | 0.000193 | 2000000 | 0.8 (0.65 - 0.95) |
| ACN | µl | 0.000108 | 2000000 | 0.67 (0.65 - 0.95) |
| FAI | mg | 0.0014 | 5000000 | 0.85 (0.65 - 0.95) |
| MABr | mg | 0.000962 | 500000 | 0.85 (0.65 - 0.95) |
| MAI | mg | 0.000829 | 1000000 | 0.85 (0.65 - 0.95) |
| PbI2 | mg | 0.00102 | 50000 | 0.83 (0.65 - 0.95) |
| DMF | ul | 5.07E-05 | 18000000 | 0.82 (0.65 - 0.95) |
| FAI | mg | 0.0014 | 5000000 | 0.85 (0.65 - 0.95) |
| IPA | ul | 5.55E-05 | 2000000 | 0.66 (0.65 - 0.95) |
| MAI | mg | 0.000829 | 1000000 | 0.85 (0.65 - 0.95) |
| PbI2 | mg | 0.00102 | 50000 | 0.83 (0.65 - 0.95) |
| CsI | mg | 0.0104 | 10000 | 0.82 (0.65 - 0.95) |
| DMF | ul | 5.07E-05 | 18000000 | 0.82 (0.65 - 0.95) |
| DMSO | ul | 7.06E-05 | 18000000 | 0.74 (0.65 - 0.95) |
| FAI | mg | 0.0014 | 5000000 | 0.85 (0.65 - 0.95) |
| MABr | mg | 0.000962 | 500000 | 0.85 (0.65 - 0.95) |
| $PbBr_2$ | mg | 0.0284 | 25000 | 0.86 (0.65 - 0.95) |
| $PbI_2$ | mg | 0.00102 | 50000 | 0.83 (0.65 - 0.95) |
| Anisole | µl | 0.000775 | 18000000 | 0.78 (0.65 - 0.95) |
| HTL Solar 4 | mg | 0.00183 | 1000000 | 0.82 (0.65 - 0.95) |
| CB | ul | 0.000107 | 2000000 | 0.67 (0.65 - 0.95) |
| FK 209 Co(III) PF6 salt | mg | 0.0429 | 5000 | 0.82 (0.65 - 0.95) |
| LiTFSi | mg | 0.00454 | 50000 | 0.84 (0.65 - 0.95) |

| | | | | |
|---|---|---|---|---|
| Spiro | mg | 0.269 | 5000 | 0.82 (0.65 - 0.95) |
| TBP | mg | 0.00352 | 500000 | 0.82 (0.65 - 0.95) |
| Ethyl cellulose | mg | 0.000667 | 1000000 | 0.92 (0.65 - 0.95) |
| Graphite | mg | 2.66E-05 | 12000000 | 0.74 (0.65 - 0.95) |
| PGMEA | ul | 3.65E-05 | 4000000 | 0.66 (0.65 - 0.95) |
| Ag paste | g | 2.35 | 1000 | 0.9 (0.85 - 0.95) |
| Au pellets | g | 73 | 100 | 0.9 (0.85 - 0.95) |
| Adhesive | m2 | 20.9 | 157 | 0.9 (0.85 - 0.95) |
| Barrier film | m2 | 46.3 | 130 | 0.9 (0.85 - 0.95) |

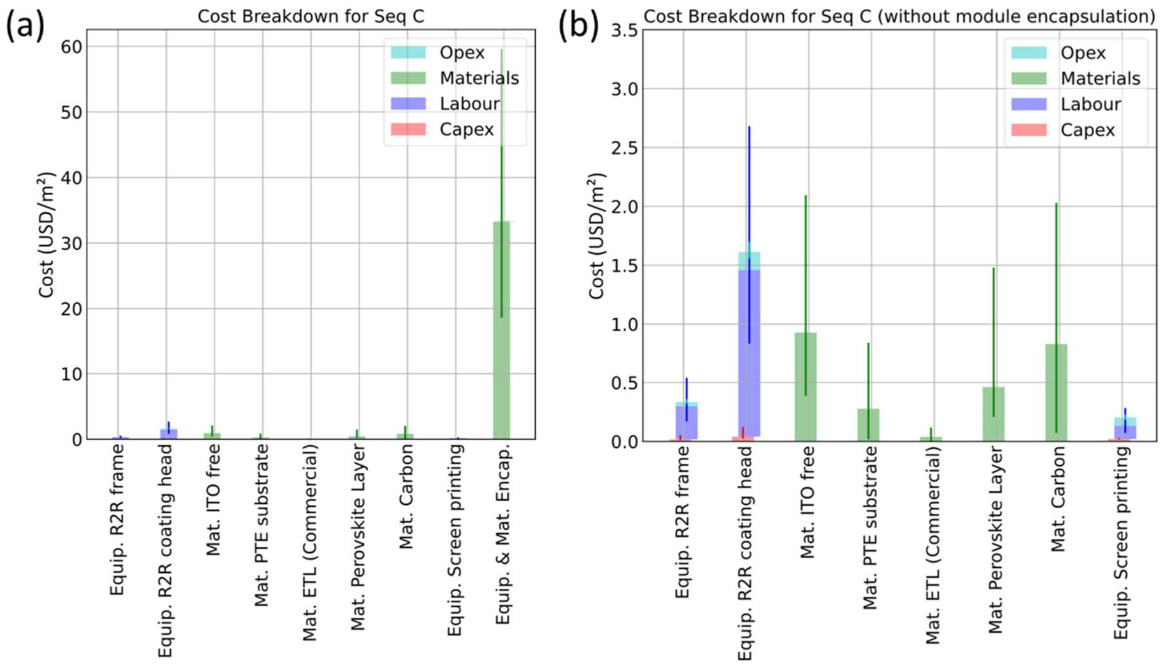


**Figure S12**. Cost breakdown for seq. C (a) with and (b) without encapsulation. The calculated total costs (median value) of seq. C with and without encapsulation are ~38 and~ 4.7 USD m$^{-2}$, respectively.

## References


1. Yoon, J. *et al.* Superflexible, high-efficiency perovskite solar cells utilizing graphene electrodes: towards future foldable power sources. *Energy Environ Sci* **10**, 337–345 (2017).
2. Feng, J. *et al.* Record Efficiency Stable Flexible Perovskite Solar Cell Using Effective Additive Assistant Strategy. *Advanced Materials* **30**, 1801418 (2018).
3. Huang, K. *et al.* High-Performance Flexible Perovskite Solar Cells via Precise Control of Electron Transport Layer. *Adv Energy Mater* **9**, 1901419 (2019).

4. Meng, X. *et al.* Bio-inspired vertebral design for scalable and flexible perovskite solar cells. *Nat Commun* **11**, 3016 (2020).
5. Wu, S. *et al.* Low-Bandgap Organic Bulk-Heterojunction Enabled Efficient and Flexible Perovskite Solar Cells. *Advanced Materials* **33**, 2105539 (2021).
6. Li, M. *et al.* Multifunctional succinate additive for flexible perovskite solar cells with more than 23% power-conversion efficiency. *The Innovation* **3**, 100310 (2022).
7. Dou, B. *et al.* Roll-to-Roll Printing of Perovskite Solar Cells. *ACS Energy Lett* **3**, 2558–2565 (2018).
8. Kim, Y. Y. *et al.* Roll-to-roll gravure-printed flexible perovskite solar cells using eco-friendly antisolvent bathing with wide processing window. *Nat Commun* **11**, 5146 (2020).
9. Heo, Y. J. *et al.* Printing-friendly sequential deposition via intra-additive approach for roll-to-roll process of perovskite solar cells. *Nano Energy* **41**, 443–451 (2017).
10. Zuo, C., Vak, D., Angmo, D., Ding, L. & Gao, M. One-step roll-to-roll air processed high efficiency perovskite solar cells. *Nano Energy* **46**, 185–192 (2018).
11. Kim, J. E. *et al.* Humidity-Tolerant Roll-to-Roll Fabrication of Perovskite Solar Cells via Polymer-Additive-Assisted Hot Slot Die Deposition. *Adv Funct Mater* **29**, 1809194 (2019).
12. Burkitt, D. *et al.* Roll-to-roll slot-die coated P–I–N perovskite solar cells using acetonitrile based single step perovskite solvent system. *Sustain Energy Fuels* **4**, 3340–3351 (2020).
13. Sutherland, L. J. *et al.* Vacuum-Free and Solvent-Free Deposition of Electrodes for Roll-to-Roll Fabricated Perovskite Solar Cells. *Adv Energy Mater* **12**, 2202142 (2022).
14. Beynon, D. *et al.* All-Printed Roll-to-Roll Perovskite Photovoltaics Enabled by Solution-Processed Carbon Electrode. *Advanced Materials* **35**, 2208561 (2023).
15. Kim, Y. Y. *et al.* Roll-to-roll gravure-printed flexible perovskite solar cells using eco-friendly antisolvent bathing with wide processing window. *Nat Commun* **11**, 5146 (2020).
16. Dou, B. *et al.* Roll-to-Roll Printing of Perovskite Solar Cells. *ACS Energy Lett* **3**, 2558–2565 (2018).
17. Vak, D. *et al.* Reverse gravure coating for roll-to-roll production of organic photovoltaics. *Solar Energy Materials and Solar Cells* **149**, 154–161 (2016).
18. Troughton, J. *et al.* Rapid processing of perovskite solar cells in under 2.5 seconds. *J Mater Chem A Mater* **3**, 9123–9127 (2015).
19. Anyi, M. *et al.* A hole-conductor–free, fully printable mesoscopic perovskite solar cell with high stability. *Science (1979)* **345**, 295–298 (2014).
20. Chu, Q. Q. *et al.* Cost effective perovskite solar cells with a high efficiency and open-circuit voltage based on a perovskite-friendly carbon electrode. *J Mater Chem A Mater* **6**, 8271–8279 (2018).
21. Zhang, F. *et al.* Structure engineering of hole-conductor free perovskite-based solar cells with low-temperature-processed commercial carbon paste as cathode. *ACS Appl Mater Interfaces* **6**, 16140–16146 (2014).
22. Liu, Z. *et al.* 15% efficient carbon based planar-heterojunction perovskite solar cells using a TiO2/SnO2 bilayer as the electron transport layer. *J Mater Chem A Mater* **6**, 7409–7419 (2018).

23. Zhou, H. *et al.* Low-temperature processed and carbon-based ZnO/CH 3 NH 3 PbI 3 /C planar heterojunction perovskite solar cells. *Journal of Physical Chemistry C* **119**, 4600–4605 (2015).
24. Babu, V. *et al.* Improved Stability of Inverted and Flexible Perovskite Solar Cells with Carbon Electrode. *ACS Appl Energy Mater* **3**, 5126–5134 (2020).
25. Hou, Y. *et al.* Inverted, Environmentally Stable Perovskite Solar Cell with a Novel Low-Cost and Water-Free PEDOT Hole-Extraction Layer. *Adv Energy Mater* **5**, 1500543 (2015).
26. Nguyen, T. L. *et al.* Semi-crystalline photovoltaic polymers with efficiency exceeding 9% in a ~300 nm thick conventional single-cell device. *Energy Environ Sci* **7**, 3040–3051 (2014).
27. Ko, S. J. *et al.* Photocurrent Extraction Efficiency near Unity in a Thick Polymer Bulk Heterojunction. *Adv Funct Mater* **26**, 3324–3330 (2016).
28. Song, S. *et al.* Hot slot die coating for additive-free fabrication of high performance roll-to-roll processed polymer solar cells. *Energy Environ Sci* **11**, 3248–3255 (2018).
29. Koh, C. W. *et al.* Enhanced Efficiency and Long-Term Stability of Perovskite Solar Cells by Synergistic Effect of Nonhygroscopic Doping in Conjugated Polymer-Based Hole-Transporting Layer. *ACS Appl Mater Interfaces* **9**, 43846–43854 (2017).
30. Ng, L. W. T. *et al.* Conformal Printing of Graphene for Single- and Multilayered Devices onto Arbitrarily Shaped 3D Surfaces. *Adv Funct Mater* **29**, 1807933 (2019).
31. Goldschmidt, A. & Streitberger, H.-J. *BASF handbook on basics of coating technology*. (William Andrew, 2003).
32. Yang, M. *et al.* High efficient and long-time stable planar heterojunction perovskite solar cells with doctor-bladed carbon electrode. *J Power Sources* **424**, 61–67 (2019).
33. Chang, N. L. *et al.* Manufacturing cost and market potential analysis of demonstrated roll-to-roll perovskite photovoltaic cell processes. *Solar Energy Materials and Solar Cells* **174**, 314–324 (2018).
34. Sutherland, L. J., Weerasinghe, H. C. & Simon, G. P. A Review on Emerging Barrier Materials and Encapsulation Strategies for Flexible Perovskite and Organic Photovoltaics. *Adv Energy Mater* **11**, 2101383 (2021).
35. Bernardo, G., Lopes, T., Lidzey, D. G. & Mendes, A. Progress in Upscaling Organic Photovoltaic Devices. *Adv Energy Mater* **11**, 2100342 (2021).
