# Supplementary material for: The first demonstration of entirely roll-to-roll fabricated perovskite solar cell modules under ambient room conditions
Source: Nat Commun. 2024 Mar 12;15:1656. doi: 10.1038/s41467-024-46016-1 (PMC10933357; doi:10.1038/s41467-024-46016-1)
Supplement: Supplementary file 3 — Reporting Summary [file 41467_2024_46016_MOESM3_ESM.pdf]

Corresponding author(s): Jacek J. Jasieniak, Tawfique Hasan, and Doojin Vak

Solar Cells Reporting Summary

Nature Portfolio wishes to improve the reproducibility of the work that we publish. This form is intended for publication with all accepted papers reporting the characterization of photovoltaic devices and provides structure for consistency and transparency in reporting. Some list items might not apply to an individual manuscript, but all fields must be completed for clarity.

For further information on Nature Research policies, including our data availability policy, see Authors & Referees.

Experimental design

Please check the following details are reported in the manuscript, and provide a brief description or explanation where applicable.

1. Dimensions

Area of the tested solar cells

☒ Yes — 0.025 cm^{-2} and 0.08 cm^{-2} for small cells. 49.5 cm^{-2} for modules
☐ No — *Explain why this information is not reported/not relevant.*

Method used to determine the device area

☒ Yes — The area of small cells were defined by optical aperture and the area of module was defined by measured active area (9.9 cm^{-2} x 5 cells). Further details are available in the Characterisation Methods.
☐ No — *Explain why this information is not reported/not relevant.*

2. Current-voltage characterization

Current density-voltage (J-V) plots in both forward and backward direction

☒ Yes — Characterisation Methods section
☐ No

Voltage scan conditions

☒ Yes — -0.2 V to 1.2 V with 20 mV step for cells (~250 mV sec-1) and -0.2 V to 5 V with 200 mV step (~1 V sec-1) for modules
☐ No — *Explain why this information is not reported/not relevant.*

Test environment

☒ Yes — Ambient condition without temperature control for all testings except the data for Fig 4c-g that was tested in a nitrogen-filled box.
☐ No — *Explain why this information is not reported/not relevant.*

Protocol for preconditioning of the device before its characterization

☒ Yes — Devices were measured as soon as prepared or kept in a dry box a day or two before the first measurement.
☐ No — *Explain why this information is not reported/not relevant.*

Stability of the J-V characteristic

☒ Yes — Maximum power point tracking was performed and the result is shown in Fig. S6d. The cell showed somewhat slow response with increased
☐ No — *Explain why this information is not reported/not relevant.*

3. Hysteresis or any other unusual behaviour

Description of the unusual behaviour observed during the characterization

☒ Yes — The perovskite cells and modules showed some hysteresis behavior.
☐ No — *Explain why this information is not reported/not relevant.*

Related experimental data

☒ Yes — Fig. 4b, Fig. 4g, Fig 5d and Fig. S6
☐ No — *Explain why this information is not reported/not relevant.*

4. Efficiency

External quantum efficiency (EQE) or incident photons to current efficiency (IPCE)

☒ Yes — A commercial IPCE setup (Peccel S20, DC mode) was used
☐ No — *Explain why this information is not reported/not relevant.*

A comparison between the integrated response under the standard reference spectrum and the response measure under the simulator

☒ Yes — Main text describing Fig. 4
☐ No — *Explain why this information is not reported/not relevant.*

For tandem solar cells, the bias illumination and bias voltage used for each subcell	☐ Yes ☒ No	*Provide a description of the measurement conditions.*<br>N/A

5. Calibration

Light source and reference cell or sensor used for the characterization	☒ Yes ☐ No	Enlitech SS-F5-3A AAA+ solar simulator for small cells and Newport Oriel 1 KW solar simulator for modules. A Si reference cell with KG-1 filter provided by Enlitech was used.<br>*Explain why this information is not reported/not relevant.*
Confirmation that the reference cell was calibrated and certified	☒ Yes ☐ No	The reference was calibrated and certified by the supplier Enlitech.<br>*Explain why this information is not reported/not relevant.*
Calculation of spectral mismatch between the reference cell and the devices under test	☒ Yes ☐ No	spectral mismatch factor with carbon-based cells was 0.92 (i.e. underestimation) but measured current densities without correction using the mismatch factor are reported.<br>*Explain why this information is not reported/not relevant.*

6. Mask/aperture

Size of the mask/aperture used during testing	☒ Yes ☐ No	0.025 cm^{-2} (circular hole with 1.8 mm diameter)<br>*Explain why this information is not reported/not relevant.*
Variation of the measured short-circuit current density with the mask/aperture area	☐ Yes ☒ No	*Report the difference in the short-circuit current density values measured with the mask and aperture area.*<br>The design of cells and modules allowed only one way or another. No comparison could be done.

7. Performance certification

Identity of the independent certification laboratory that confirmed the photovoltaic performance	☐ Yes ☒ No	*Identify the independent certification laboratory.*<br>We are currently in the process of developing an encapsulation technique specifically tailored for flexible PeSC modules. As a result, we are unable to send the modules to external organizations for testing at this time.<br><br>The small cells utilised in our study possess a unique design that necessitates specialized automated testing. Conducting tests on these cells by a third party would require significant modifications to the layout and process.
A copy of any certificate(s)	☐ Yes ☒ No	*Certificate copies should be provided in the Supplementary information. Please state the supplementary item number.*<br>N/A

8. Statistics

Number of solar cells tested	☒ Yes ☐ No	Figure captions and main text for Fig 3 and Fig 4<br>*Explain why this information is not reported/not relevant.*
Statistical analysis of the device performance	☒ Yes ☐ No	Figure captions and main text for Fig 3 and Fig 4<br>*Explain why this information is not reported/not relevant.*

9. Long-term stability analysis

Type of analysis, bias conditions and environmental conditions	☐ Yes ☒ No	*Provide a description of the type of analysis, bias conditions and environmental conditions (e.g. illumination type, temperature, atmosphere humidity, encapsulation method, preconditioning temperature, bias) for each long-term stability analysis carried out; see ref. 7 and 8 for details.*<br>The encapsulation technique for the flexible PeSC modules is being developed. The stability testing would be done in the fugure.
